# Supplementary material for: Effects of RAGE Deletion on the Cardiac Transcriptome during Aging
Source: Int J Mol Sci. 2022 Sep 22;23(19):11130. doi: 10.3390/ijms231911130 (PMC9569842; doi:10.3390/ijms231911130)
Supplement: Supplementary file 1 [file ijms-23-11130-s001.zip › Supplementary Data.pdf]

Supplementary Data

Effects of RAGE Deletion on the Cardiac Transcriptome during Aging

Francesco Scavello [1,†], Luca Piacentini [2,†], Stefania Castiglione [1], Filippo Zeni [1], Federica Macrì [1], Manuel Casaburo [3], Maria Cristina Vinci [4], Gualtiero I. Colombo [5,*] and Angela Raucci [1,3,*]

[1] Unit of Experimental Cardio-Oncology and Cardiovascular Aging, Centro Cardiologico Monzino IRCCS, 20138 Milan, Italy
[2] Bioinformatics and Artificial Intelligence Facility, Centro Cardiologico Monzino IRCCS, 20138 Milan, Italy
[3] Animal Facility, Centro Cardiologico Monzino IRCCS, 20138 Milan, Italy
[4] Vascular Biology and Regenerative Medicine Unit, Centro Cardiologico Monzino IRCCS, 20138 Milan, Italy
[5] Unit of Immunology and Functional Genomics, Centro Cardiologico Monzino IRCCS, 20138 Milan, Italy
* Correspondence: gualtiero.colombo@cardiologicomonzino.it (G.I.C.); araucci@ccfm.it (A.R.);
Tel.: +39-025-800-2464 (G.I.C.); +39-025-800-2802 (A.R.); Fax: +39-025-800-2342 (G.I.C. & A.R.)
† These authors contributed equally to this work.

Table S3. Sequences of mouse forward and reverse primers used for RT-qPCR

Gene	Forward	Reverse
Adgrf5	5’-GGAAGAACAGGACATCCGCTCA-3’	5’-CCAGGAGTTCAAGGCAGACTTG-3’
Igtp	5’-AGCCCGTCTTTTCACGACTT-3’	5’-CTCCAGGTTGGCAGTGTCAT-3’
Irgm2	5’-GGCAGTTGAGTCACCTGAGG-3’	5’-CCCCTTCTTTCACGGCAGT-3’
Ifit3	5’-CGAGCAAAAATGTGCTTTGA-3’	5’-GCTCCCCTTCAGCTTCTTCT-3’
P2ry1	5’-CCTGCTATGACACCACGTCCAA-3’	5’-AGCGGAGAGTTGTCCAGGTCAT-3’
Rgs4	5’-CTGAAGTCGGAATACAGCGAGG-3’	5’-CTGGTGCAAGAGTCCAGGTTCA-3’
Hmgcs2	5’-TGCTATGCAGCCTACCGCAAGA-3’	5’-GCCAGGGATTTCTGGACCATCT-3’
Rps24	5’-TGGTGGCAAGACCACTGGCTTT-3’	5’-CTGTTCTTGCGTTCCTTTCGCTG-3’
C4a	5’-GGAGAGTGGAACCTGTAGACAG-3’	5’-CACTCGAACACGAGTTGGCTTG-3’
Fitm2	5’-TGCATGAGGTGAAGACGGAc-3’	5’-AGTGTCCTTGAGGCATTGGG-3’
Hprt	5’-GGAGCGGTAGCACCTCCT-3’	5’-CCAAATCCTCGGCATAATGA-3’
Ldha	5’-AGACAAACTCAAGGGCGAGA-3’	5’-CAGCTTGCAGTGTGGACTGT-3’

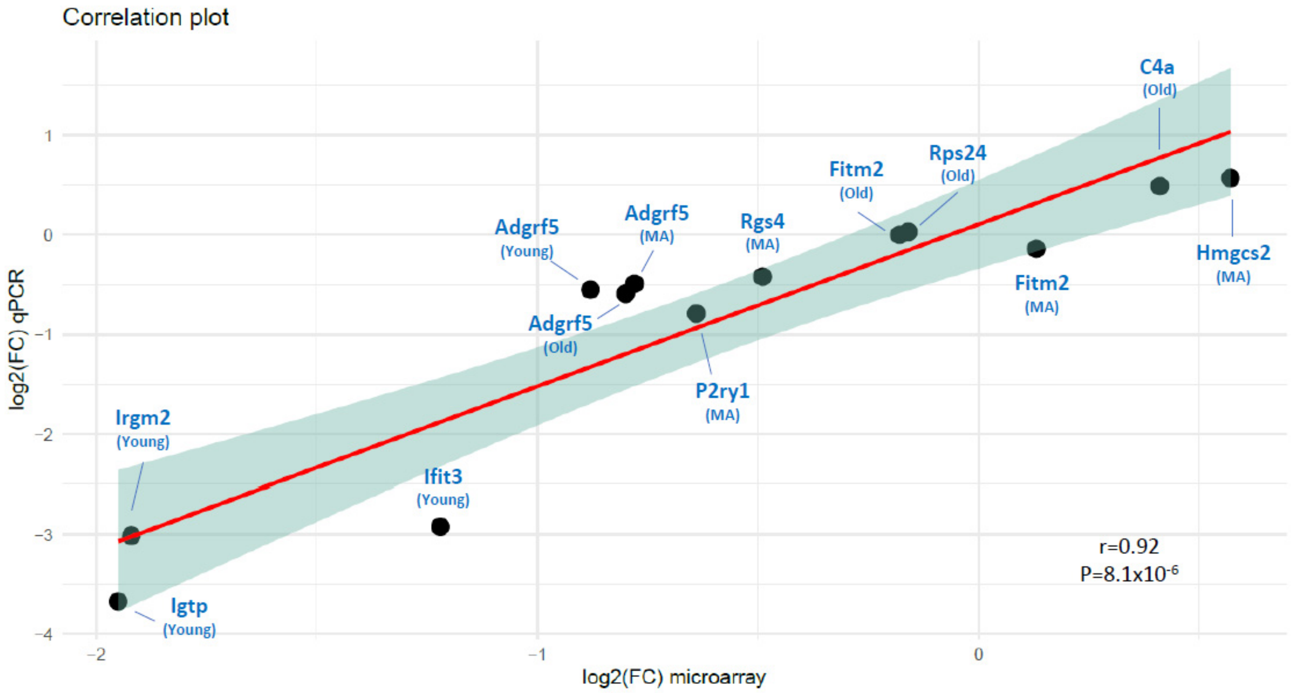

Figure S1. Correlation plot. Technical validation by RT-qPCR of 10 selected genes (*Igtp*, *Irgm2*, *Ifit3*, *Adgrf5*, *P2ry1*, *Rgs4*, *Fitm2*, *Rps24*, *C4a*, and *Hmgcs2*) differentially expressed in at least one of the comparisons between *Rage-/-* vs. WT in Young, MA or Old mice (*cf.* in brackets). Pearson's correlation coefficient (r) was computed to assess the degree of association between the $\log_2$ mean fold differences (log2FC) of these genes tested by microarray (x-axis) and RT-qPCR (y-axis). The trendline is colored in red whereas the 95% confidence interval of the trendline is depicted in light green.
